# Data on the effects of remote ischemic preconditioning in the lungs after one-lung ventilation^☆^

**DOI:** 10.1016/j.dib.2018.09.085

**Published:** 2018-10-04

**Authors:** Astrid Bergmann, Christian Breitling, Göran Hedenstierna, Anders Larsson, Moritz Kretzschmar, Alf Kozian, Thomas Hachenberg, Thomas Schilling

**Affiliations:** a Department of Anesthesiology and Intensive Care Medicine, Otto-von-Guericke-University Magdeburg, Germany; b Department of Medical Sciences, Hedenstierna Laboratory, Uppsala University, Sweden; c Hedenstierna Laboratory, Department of Medical Sciences, Uppsala University, Sweden; d Hedenstierna Laboratory, Department of Surgical Sciences, Uppsala University, Sweden

**Keywords:** Animal model, One-lung ventilation, Remote ischemic preconditioning, Diffuse alveolar damage, Pulmonary inflammatory response

## Abstract

This article contains data on experimental endpoints of a randomized controlled animal trial. Fourteen healthy piglets underwent mechanical ventilation including injurious one-lung ventilation (OLV), seven of them experienced four cycles of remote ischemic preconditioning (RIP) on one hind limb immediately before OLV, seven of them did not receive RIP and served as controls, in a randomized manner. The two major endpoints were (1) pulmonary damage assessed with the diffuse alveolar damage (DAD) score and (2) the inflammatory response assessed by cytokine concentrations in serum and in bronchoalveolar lavage fluids (BAL). The cytokine levels in the homogenized lung tissue samples are presented in the original article. Further interpretation and discussion of these data can be found in Bergmann et al. (in press).

**Specifications table**

**Table:** 

Subject area	*Randomized, controlled animal experiment, medicine*
More specific subject area	*Respiratory Physiology*
Type of data	*Table, images (microscopy), text file, graphs, figures*
How data was acquired	*Experimental protocol, cytokine and protein measurement, microscopy*
Data format	*Analyzed*
Experimental factors	*Prospective, randomized, controlled experimental trial in pigs*
Experimental features	*The effects of remote ischemic preconditioning (RIP) on the lungs have been examined in pigs after mechanical ventilation including one-lung ventilation. Respiratory and hemodynamic parameters have been collected, and lung damage in terms of the diffuse-alveolar-damage score (DAD score) was assessed in the harvested lungs by staining and microscopy of the pulmonary tissue accompanied by cytokine expression*
Data source location	*University of Uppsala, Sweden*
	*Otto-von-Guericke-University of Magdeburg, Germany*
Data accessibility	*Data with this article*

**Value of the data**

• The data presented in this animal study open the way to further research on ischemic preconditioning in clinical studies. The data provide valuable information on the effects of RIP on the lung, especially concerning DAD scores which are not possible to obtain in clinical settings as the lungs of patients cannot be histologically examined in terms of harvesting standardized parts of the organ.
• The development of cytokines in serum and in BAL fluid confirm that an immunological response to RIP takes place which shows a different time pattern in each cytokine, depending on the site of measurement as well (either serum or lungs). This is a field for further research in terms of longer protocols after RIP and of identifying cytokines in other tissues, e.g. muscle.
• The data open the field for examining early stages in cytokine development, i.e. assessing the mRNA of cytokines in different tissues.
• These data might support RIP to become a tool that is easily applied and cheap to improve patients’ outcome after thoracic surgery and might be considered in the clinical setting.

## 1 Data

The data of a randomized animal experiment on the effects of remote ischemic preconditioning (RIP) on alveolar integrity and the pulmonary alveolar pro-inflammatory response after injurious one-lung ventilation (OLV) are shared with this data article.

The diffuse alveolar damage score was used to determine the effects of injurious mechanical ventilation in a porcine model of OLV [2], [3]. In addition, the effect of RIP on alveolar injury was estimated by DAD. The DAD data are presented in Table 1; separately for the left lung that was blocked during OLV with an Arndt bronchial blocker, and for the right lung which was ventilated throughout the whole experiment. The RIP group comprised seven subjects (n=7) as well as the control group (n=7). Please note that, despite the lack of differences in the total DAD scores between both lungs and between the groups, the features “alveolar edema” and “microhemorrhage” were more pronounced in the right (ventilated) lungs of the RIP pigs.

**Table 1:** Diffuse Alveolar Damage Score (DAD) of the left lungs (that were blocked during OLV) and right lungs (that were ventilated) of the RIP group (n=7) and control animals (n=7), presented as medians and interquartile ranges (IQR, P_25_-P_75_) (*=p<0.05 in comparison of RIP pigs with controls).

Lung	**Alveolar Edema**		**Interstitial Edema**		**Microhemorrhage**	
	**Control**	**RIP**	**Control**	**RIP**	**Control**	**RIP**
left	6.7 (6.1–7.6)	5.9 (5.2–6.5)	4.6 4.4–5.2)	4.9 (4.4–5.7)	4.1 (3.9–4.3)	4.3 (3.9–5.5)
right	5.4 (4.5–5.7)	6.0⁎ (5.5–6.5)	4.7 (4.3–4.9)	5.1 (4.3–5.5)	3.5 (2.4–4.4)	4.8⁎ (4.5–5.4)

	**Neutrophil Infiltration**		**Alveolar Overtistension**		**Microatelectasis**		**DAD-Score**	
Lung	**Control**	**RIP**	**Control**	**RIP**	**Control**	**RIP**	**Control**	**RIP**
left	7.4 (5.7–8.0)	5.6 (4.7–7.4)	5.9 (5.1–6.3)	5.0 (4.0–6.8)	1.9 (1.6–2.5)	2.3 (1.7–3.6)	29.8 (28.3–32.1)	30.4 (26.5–32.6)
right	6.2 (5.6–7.0)	5.6 (4.9–7.2)	5.3 (4.4–6.6)	4.8 (4.4–6.2)	1.5 (1.4–2.6)	2.5 (1.8–3.3)	29.3 (26.8–31.3)	30.0 (28.2–31.6)

**Fig. 1:**
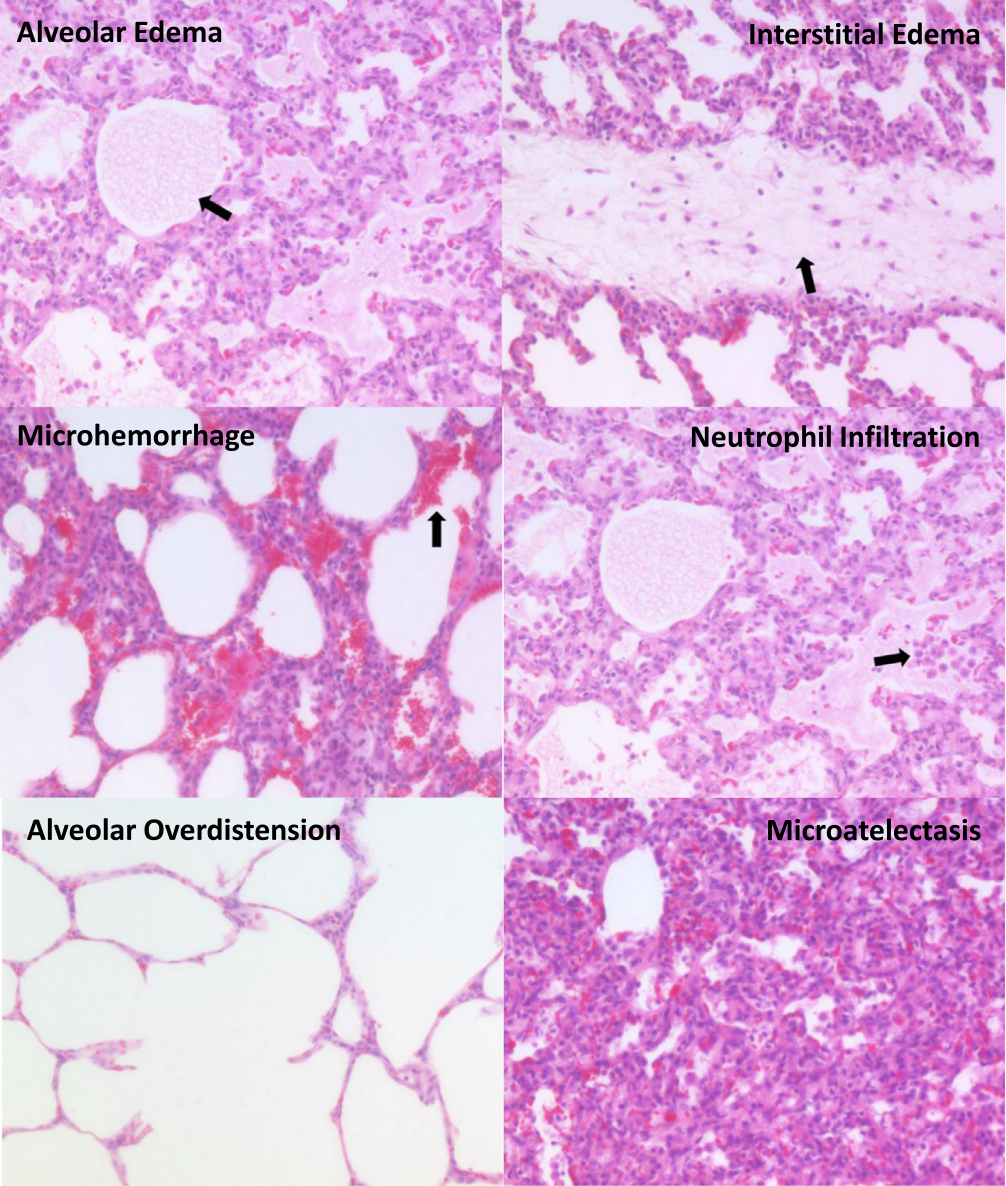
Representative images from a single piglet that illustrate the characteristics of the diffuse alveolar damage score (H&E staining, x 100).

The histological images representing the typical consequences of mechanical ventilation-induced pulmonary injury that form the basis for the DAD score are presented in Fig. 1. For each characteristic element of DAD (alveolar and interstitial edema, microhemorrhage, neutrophil infiltration, alveolar overdistension and microatelectasis), a representative image from a single piglet was displayed.

**Fig. 2:**
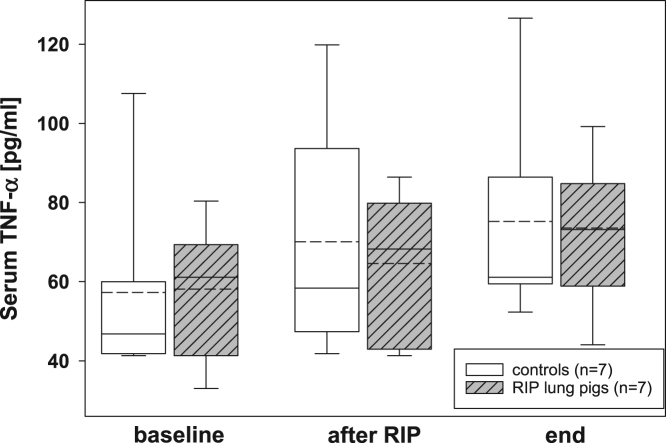
Serum TNF-α concentrations in the control group (n=7) and in RIP pigs (n=7) at baseline, immediately after the RIP procedure before OLV was started, and at the end of the experiment.

**Fig. 3:**
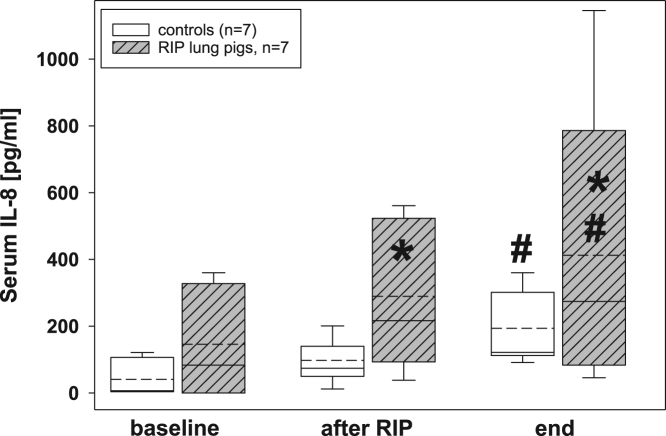
Serum IL-8 concentrations in the control group (n=7) and in RIP pigs (n=7) at baseline, immediately after the RIP procedure before OLV was started, and at the end of the experiment. *=p<0.05 in comparison of RIP pigs with controls; ♯=p<0.05 within groups when compared to baseline.

**Fig. 4:**
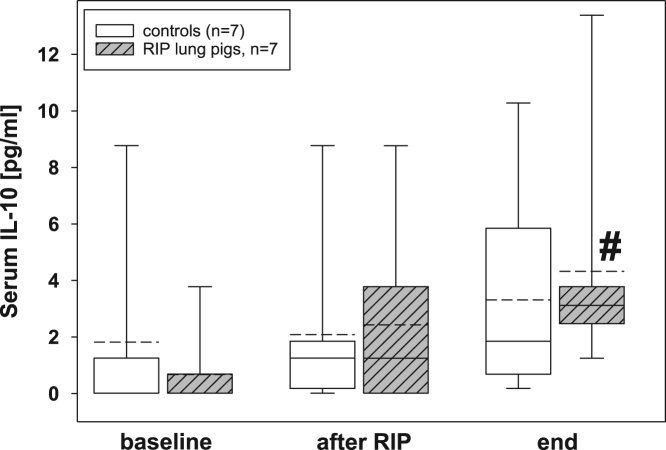
Serum IL-10 concentrations in the control group (n=7) and in RIP pigs (n=7) at baseline, immediately after the RIP procedure before OLV was started, and at the end of the experiment. *=p<0.05 in comparison of RIP pigs with controls; ♯=p<0.05 within groups when compared to baseline.

**Fig. 5:**
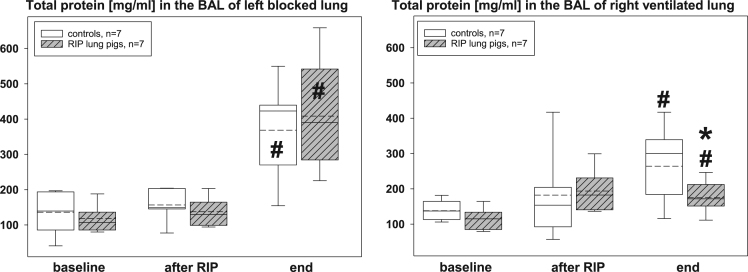
Total protein concentrations [mg/ml] in the bronchoalveolar lavage fluids (BAL) of controls (n=7) and RIP lung pigs (n=7), separately displayed for the left blocked and right ventilated lungs at baseline, immediately after the RIP procedure before OLV was started, and at the end of the experiment. *=p<0.05 in comparison of RIP pigs with controls; ♯=p<0.05 within groups when compared to baseline.

**Fig. 6:**
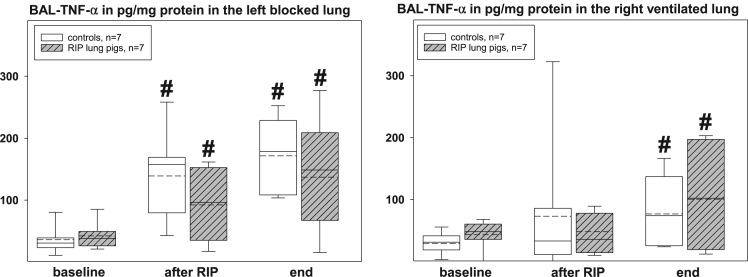
TNF-α concentrations in the lavage fluids (BAL) of controls (n=7) and RIP pigs (n=7), separately displayed for the left, blocked and right, ventilated lungs at baseline, immediately after the RIP procedure before OLV was started, and at the end of the experiment. ♯=p<0.05 within groups when compared to baseline.

**Fig. 7:**
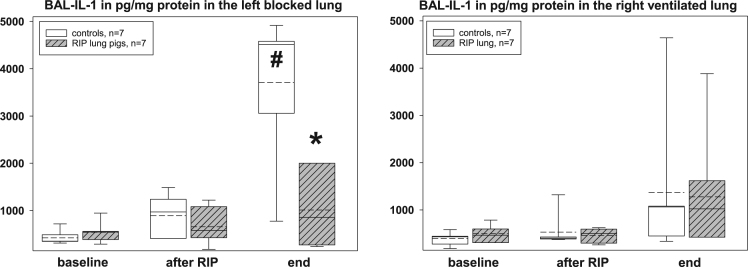
Interleukin-1β concentrations in the lavage fluids (BAL) of controls (n=7) and RIP pigs (n=7), separately given for the left, blocked and right, ventilated lungs at baseline, immediately after the RIP procedure before OLV was started, and at the end of the experiment. *=p<0.05 in comparison of RIP pigs with controls; ♯=p<0.05 within groups when compared to baseline.

**Fig. 8:**
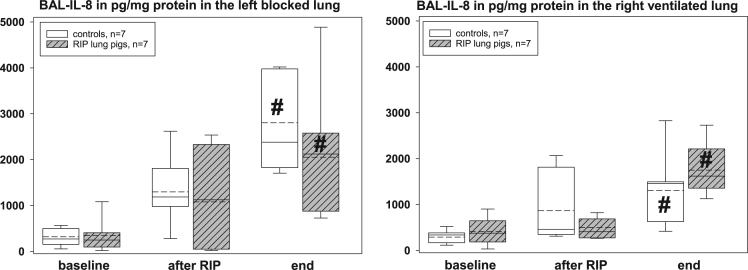
Interleukin-8 concentrations in the lavage fluids (BAL) of controls (n=7) and RIP pigs (n=7), separately given for the left, blocked and right, ventilated lungs at baseline, immediately after the RIP procedure before OLV was started, and at the end of the experiment. ♯=p<0.05 within groups when compared to baseline.

**Fig. 9:**
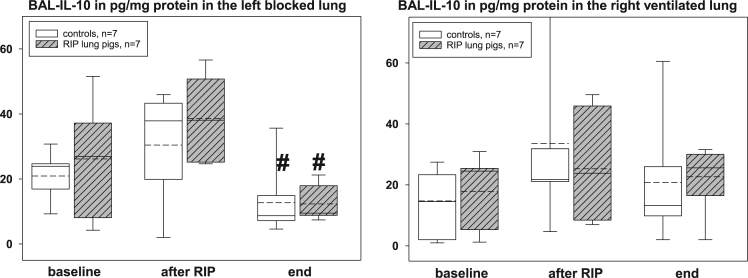
Interleukin-10 concentrations in the lavage fluids (BAL) of controls (n=7) and RIP pigs (n=7), separately given for the left, blocked and right, ventilated lungs at baseline, immediately after the RIP procedure before OLV was started, and at the end of the experiment. ♯=p<0.05 within groups when compared to baseline.

The presented histopathological changes of lung tissue as a consequence of one-lung ventilation and RIP were accompanied by expression of different pro- and anti-inflammatory mediators. The following figures highlight the time courses of cytokine release into serum samples and bronchoalveolar lavage fluids and lung tissues – levels, separately displayed for controls (n=7) and piglets previously treated with the RIP procedure (Fig. 2, Fig. 3, Fig. 4, Fig. 5, Fig. 6, Fig. 7, Fig. 8, Fig. 9).

## 2 Experimental design, materials and methods

Mechanical ventilation, especially one-lung ventilation (OLV) is detrimental for the lung [6]. Especially in thoracic surgery, though, OLV is essential for making the surgery on the lung possible [5]. Many attempts have been made to find a way to attenuate this pulmonary damage which might be detrimental for the postoperative outcome for patients that underwent thoracic surgery. RIP seems to have beneficial effects in terms of alleviating the ischemia-reperfusion injury in several organs, mainly in the heart [4]. As the procedure is cheap and easy to perform, a protocol was designed to analyze the effects of RIP on the lungs of healthy piglets that experienced mechanical ventilation including OLV. The present experimental design, materials and methods are in accordance with the corresponding experiment ([1], in press). It was scheduled as a prospective, controlled, animal trial in a single cohort of juvenile piglets. The Animal Ethics Committee of Uppsala University (Sweden) approved the experimental protocol.

**Fig. 10:**
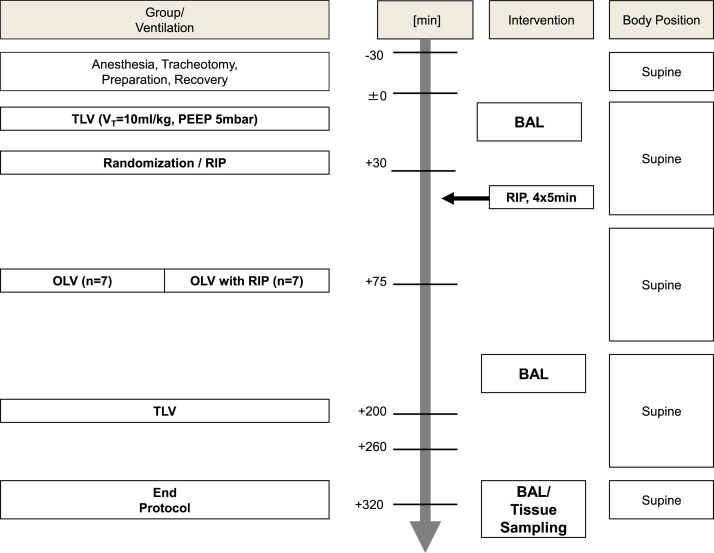
Timeline of the experimental protocol.

Fig. 10 illustrates the experimental protocol of the present study in a chart, indicating the time points, where interventions took place and samples were taken.

Fourteen 2.5 month-old piglets were prepared using the algorithm described in our main manuscript ([1], in press). Briefly, they were randomly assigned to either the RIP or the control group. Then, general anesthesia was induced and the airway was secured via tracheostomy. A central venous catheter was inserted in the jugular vein, an arterial line in the carotid artery and a PiCCO catheter into the femoral artery. In those pigs designated to the RIP group, RIP was performed with a blood-pressure-cuff on the left hind-limb. The cuff was inflated up to 200 mmHg and kept there for five minutes, followed by five minutes of reperfusion, this being done four times. A left-sided bronchial blocker was placed into the left main bronchus under bronchoscopic control, the balloon being inflated to ensure one-lung-ventilation of the right lung according to the flow-chart of the protocol (Fig. 10). Blood samples were taken and BAL was performed according to the timeline of the protocol. After euthanization of the animal, the lungs were harvested, samples were taken and processed for histological analysis to determine the DAD score. The measurement of cytokines from serum, BAL fluid and lung tissue was carried out according to the manufacturer׳s instructions.
