# Supplementary material for: Data on the effects of remote ischemic preconditioning in the lungs after one-lung ventilation
Source: Data Brief. 2018 Oct 4;21:441–8. doi: 10.1016/j.dib.2018.09.085 (PMC6198023; doi:10.1016/j.dib.2018.09.085)
Supplement: Supplementary file 1 — Supplementary material. [file mmc1.docx]

Astrid Bergmann, M.D., D.E.S.A.

Otto-von-Guericke-University Magdeburg

Dept. of Anaesthesiology and Intensive Care

Leipziger Str. 44

D 39120 Magdeburg

Germany

Managing Editor

Data in Brief

Magdeburg, September 11^th^ 2018

Manuscript DIB-D-18-02334
**Data on the Effects of Remote Ischemic Preconditioning**

**in the Lungs after One-Lung Ventilation**

**Authors:** Astrid Bergmann, M.D., D.E.S.A.^1*^, Christian Breitling, M.D.^2^, Göran Hedenstierna, M.D., Ph.D.^3^, Anders Larsson, M.D., Ph.D., D.E.A.A.^4^, Moritz Kretzschmar, M.D., Ph.D., D.E.S.A.^5^, Alf Kozian, M.D., Ph.D.^6^, Thomas Hachenberg, M.D., Ph.D.^7^, Thomas Schilling, M.D., Ph.D., D.E.A.A.^8^

**Affiliations:**

1. Senior Consultant in Cardiothoracic Anesthesia, Department of Anesthesiology and Intensive Care Medicine, Otto-von-Guericke-University Magdeburg, Germany, and
   Research Anesthesiologist, Department of Medical Sciences, Hedenstierna Laboratory, Uppsala University, Sweden
2. Consultant in the Department of Anesthesiology and Intensive Care Medicine, Otto-von-Guericke-University Magdeburg, Germany
3. Senior Professor in Clinical Physiology, Hedenstierna Laboratory, Department of Medical Sciences, Uppsala University, Sweden
4. Professor of Anesthesiology and Intensive Care, Hedenstierna Laboratory, Department of Surgical Sciences, Uppsala University, Sweden
5. Senior Consultant in Cardiothoracic Anesthesia, Department of Anesthesiology and Intensive Care Medicine, Otto-von-Guericke-University Magdeburg, Germany
6. Professor of Anesthesia, Department of Anesthesiology and Intensive Care Medicine, Otto-von-Guericke-University Magdeburg, Germany
7. Professor of Anesthesia and Chair of the Department of Anesthesiology and Intensive Care Medicine, Otto-von-Guericke-University Magdeburg, Germany
8. Professor of Anesthesia and Senior Consultant in Anesthesia, Department of Anesthesiology and Intensive Care Medicine, Otto-von-Guericke-University Magdeburg, Germany

The authors declare that there is no conflict of interest.
